# VSIG4 as a novel immune-related diagnostic biomarker and therapeutic target in renal fibrosis

**DOI:** 10.1016/j.clinsp.2025.100817

**Published:** 2025-11-10

**Authors:** Chen Gao, Fenghua Peng, Xubiao Xie, Longkai Peng

**Affiliations:** Department of Kidney Transplantation, The Second Xiangya Hospital of Central South University, Changsha, China

**Keywords:** Renal fibrosis, Diagnostic markers, Machine Learning, VSIG4

## Abstract

**Background:**

The aim of this study was to find novel diagnostic markers for renal fibrosis based on bioinformatics analysis.

**Methods:**

In this study, we first identified differentially expressed genes and modular genes associated with renal fibrosis. We utilized the GSE76882 dataset (81 renal fibrosis patients and 91 control samples) to identify these genes and then intersected them with the DEGs in the GSE120495 dataset (5 renal fibrosis patients and 5 control samples). To gain insights into the biological processes involved, we performed GO and KEGG analyses on the intersection genes and constructed protein interaction networks. Next, we employed the LASSO regression algorithm and the random forest algorithm to further screen the candidate biomarkers. The sensitivity and specificity of the candidate biomarkers were assessed using ROC curves. Finally, we induced renal fibrosis using the Unilateral Ureteral Obstruction (UUO) model (characterized by renal tubular damage and fibrosis in response to obstructed urinary flow) to observe the impact of the candidate biomarkers on the progression of renal fibrosis.

**Results:**

A total of 383 differential genes and 689 renal fibrosis-related module genes were identified from the GSE76882 dataset, and 213 genes were obtained by intersecting the two. A total of 2431 differentially expressed genes were identified from the GSE120495 dataset, and 34 genes were obtained by intersecting them with the results of the previous step. Five candidate biomarkers (SFN, ARHGAP9, VSIG4, ISG20, CD3G) were identified by LASSO regression analysis and random forest algorithm. The comprehensive AUC values of the five genes in both datasets were greater than 0.7. Finally, in vivo experiments were performed to verify that VSIG4 may promote renal function injury induced by UUO.

**Conclusions:**

VSIG4 is highly expressed in UUO and may be an important mediator involved in renal fibrosis.

## Highlights

• VSIG4 is highly expressed in renal fibrosis tissues.
• VSIG4 may be an important mediator involved in renal fibrosis.
• VSIG4 may be a potential biomarker for renal fibrosis.

## Introduction

Chronic Kidney Disease (CKD) is a global public health problem with high morbidity and mortality, and renal fibrosis is the most common endpoint and core pathological process of CKD.^1^^,^^2^ Renal fibrosis is a complex process, which mainly includes glomerulosclerosis, tubulointerstitial fibrosis, and intrarenal vascular sclerosis, and its pathological features are characterized by increased synthesis and decreased degradation of matrix proteins, resulting in the deposition of matrix proteins in the renal interstitium.^3^^,^^4^ The degradation of matrix proteins is affected by protease inhibitors that inactivate renal proteases, which in turn promote fibrosis.^5^ Although the pathogenesis and molecular mechanisms of renal fibrosis have been extensively researched, effective interventions are still lacking. Considering that renal fibrosis can trigger renal failure, it is particularly crucial to deepen the understanding of its novel mechanisms and search for potential therapeutic targets.

With the rapid development of sequencing technology, comprehensive bioinformatics analysis based on high-throughput sequencing has opened up an important way for studying the mechanisms of disease occurrence and developing new therapeutic strategies.^6^, ^7^, ^8^ Sun et al. used bioinformatics methods to screen diagnostic genes and revealed that knockdown of ISG20 distinctly suppressed the expression of the expressions of fibrotic proteins (α-SMA and fibronectin), indicating that ISG20 promotes the progression of renal fibrosis.^9^ Zhang et al. explored the expression patterns of methylation-related genes in renal fibrosis and constructed a five-gene diagnostic model using a machine learning algorithm to predict the incidence of patients, which is helpful for the prevention and treatment of renal fibrosis.^10^ Guo et al. identified four potential diagnostic biomarkers in the peripheral blood of patients with renal fibrosis and comprehensively analyzed their diagnostic value, drug sensitivity, and correlation with immune infiltration.^11^ However, the screening of biomarkers and their involvement in the disease process based on bioinformatics approaches remains inadequate, and further research is needed.

Therefore, this study aimed to screen for diagnostic markers of renal fibrosis and determine their involvement in the disease process. Specifically, in this study, differentially expressed genes and modular genes associated with renal fibrosis were identified from the GSE76882 dataset and intersected with DEGs from the GSE120495 dataset. GO and KEGG analyses were performed on these intersection genes to identify their associated biological processes and construct protein interaction networks. The risk genes were then further screened using the LASSO regression algorithm and the random forest algorithm, while the sensitivity and specificity of the risk genes were tested using ROC curves. Finally, renal fibrosis was induced by the UUO model to observe whether the risk genes could influence the progression of action of renal fibrosis.

## Materials and methods

### Microarray datasets acquisition

Two microarray datasets (GSE76882 and GSE120495) of renal fibrosis were downloaded from the Gene Expression Omnibus (GEO) database. GSE76882 contains 274 samples, of which 81 patients with renal fibrosis and 91 controls were selected for inclusion in this study. Five renal fibrosis patients and five control samples were selected from GSE120495. The GEO database is a public database, and the data are freely available, so ethics committee approval is not required.

### Identification of differentially expressed genes (DEGs)

The “limma” package was utilized to screen DEGs between renal fibrosis patients and control samples. An adjusted p-value of < 0.05 and log2 |Fold Change| > 1.0 were considered statistically significant. The “ggplot2” package in *R* was used to plot the volcano plot for the two groups.

### Construction of weighted gene co-expression network (WGCNA)

Gene co-expression networks were constructed by correlating gene expression levels with phenotypic characteristics to investigate potential interactions between genes. The *R* package “WGCNA” was used to construct the gene co-expression network. First, we selected the top 5000 genes with an absolute median difference from the dataset for analysis. After filtering out abnormal samples and genes, we calculated the correlation coefficient between every two genes, constructed the gene expression similarity matrix, and transformed it into the adjacency matrix. Next, the optimal soft threshold was selected to construct the scale-free network. The adjacency matrix was transformed into a Topological Overlap Matrix (TOM), and the corresponding dissimilarity was calculated. Finally, gene module detection was performed based on average hierarchical clustering and dynamic tree cutting. Module Membership (MM) and Gene Importance (GS) were calculated to assess the correlation between specific gene modules and phenotypes. Meanwhile, the intersection of DEG with the genes from the module most significantly associated with clinical features was defined as renal fibrosis-related DEGs.

### Protein-Protein interaction (PPI) network construction and analysis

To identify and assess protein functional relationships and PPI networks for renal fibrosis-related DEGs, we utilized the Search Tool for the Retrieval of Interacting Genes. The results of the STRING analysis were then imported into Cytoscape (interaction score = 0.4), which was used to select the key nodes with the strongest connectivity to visualize the molecular interaction network. The nodes with the most interactions with neighboring nodes were considered as the key nodes. The importance of each node was evaluated using CytoHubba.

### Functional and pathway enrichment analysis

To further visualize the biological function of DEGs, Gene Ontology (GO) and Kyoto Encyclopedia of Genes and Genomes (KEGG) enrichment analyses were performed in the Comparative Toxicogenomics Database. The p-value of less than 0.05 was identified as a significant term. The “ggpubr” and “ggplot2” package in *R* was used to visualize the results.

### Selection of significant diagnostic features and model construction

In the GSE76882 data, the R package “glmnet” was used for Least Absolute Shrinkage and Selection Operator (LASSO) analysis to identify the most valuable predictive genes. The parameters used for the LASSO analysis were as follows: the penalty parameter λ was determined by the minimum criteria of 10-fold cross-validation, and the lambda sequence was generated using the default settings in the “glmnet” function. The optimal λ was selected based on the smallest cross-validation error. The genes and their coefficients were then determined using the best λ-value (RMSE = 0.4658, *R*^2^ = 0.1404, and MAE = 0.429). For the Random Forest model, 10-fold cross-validation was employed, and 500 trees were used for the forest (RMSE = 0.4684, *R*^2^ = 0.1329, and MAE = 0.4088). The Area Under the Curve (AUC) from a Receiver Operating Characteristic Curve (ROC) analysis was calculated to test the diagnostic performance of each candidate gene. The *R* package “pROC” was used to draw ROC curves. The relationship between gene expression and renal fibrosis was then investigated by binomial logistic regression of generalized linear models.

### Animal experiment

Twenty C57BL/6 male mice (18 ± 2 g) were purchased from Shanghai Animal Center (Shanghai, China). The experimental protocol was approved by the Ethics Committee for Animal Experimentation of the ZhaoFenghua Biotech Company (Nanjing, China, No. IACUC-20,230,901–1). Mice were housed in a light/dark cycle for 12 h at 22 °C–24 °C and had free access to food and water. The animals were randomly divided into two groups, with ten animals in each group. The Unilateral Ureteral Obstruction (UUO)-induced renal fibrosis model was established as previously described.^12^ Briefly, the mice were anesthetized with 1 % pentobarbital (10 μL/g), the left ureter was exposed by a midline incision, and then it was obstructed by two-point ligations with 6–0 silk sutures.^13^ Blood samples were collected after the mice were sacrificed on the seventh day. The left kidney was dissected, and half of the left kidney was preserved in 10 % formalin solution and sectioned for histological examination, while the remainder was frozen in liquid nitrogen for protein extraction. No animals were excluded from the study. The animal experiments reported herein were conducted in full compliance with the Animal Research: Reporting of In Vivo Experiments (ARRIVE) guidelines, and with the National Research Council's Guide for the Care and Use of Laboratory Animals.

### Lentivirus infection

VSIG4 shRNA lentiviral particles were purchased from Santa Cruz (Santa Cruz Biotechnology, Santa Cruz, California, USA). The lentiviral vector was delivered to the kidneys of experimental mice by intrarenal injection. The VSIG4 knockout mice model was established by left kidney injecting of 85 µL purified sh-VSIG4 (3.5 × 108 TU/mL).

### Renal function test

The blood was centrifuged at 3000 rpm for 15 minutes at 4 °C to collect serum. Serum Creatinine (SCr) and Blood Urea Nitrogen (BUN) levels were examined using the Scr assay kit (C011–1, Jiangcheng Bio, Nanjing) and BUN assay kit (C013–2, Jiangcheng Bio, Nanjing) according to the manufacturer's instructions.

### Hematoxylin and eosin and masson staining

Kidney tissues were fixed with 10 % formalin for 24 h, dehydrated, and embedded in paraffin, then cut into 4 μm sections and mounted on slides. The prepared slides were deparaffinized twice in xylene and hydrated in gradient ethanol, and then stained with Hematoxylin and Eosin (HE) and Masson trichrome, respectively. The sections were imaged at 400 × magnification using an optical microscope.^14^

### Western blotting

The dissected kidney tissues were lysed by adding them to RIPA lysis buffer, followed by centrifugation of the lysate products at 12,000 rpm for 10 min at 4 °C, and the supernatant was collected to obtain the total protein solution. The protein concentration was determined using an enhanced BCA protein assay kit (Beyotime Biotechnology, China), and then the proteins were separated by 10 % SDS-PAGE (Bio-Rad, CA) and transferred to a nitrocellulose membrane. After being blocked with 5 % BSA for 1 h, the blots were incubated with primary antibody (anti-*VSIG4*, anti-collagenIII, anti-FN) at 4 °C overnight. Subsequently, the blots were washed three times with 1 × TBST and incubated with secondary antibody at room temperature for 2 h. Finally, the membranes were scanned using the Odyssey® CLx imaging system (LI-COR Biosciences, USA) and the density of the bands was determined using ImageJ software.

### Immunohistochemistry

Antigen retrieval was performed on processed kidney tissue sections, and the tissues were stained immunohistochemically according to the instructions in the kit. Briefly, the sections were deparaffinized with xylene, rehydrated in graded ethanol, and endogenous peroxidase was blocked with 3 % H_2_O_2_ for 15 min at room temperature. The sections were then incubated in recovery buffer and boiled for 5 min. After washing three times with PBS, the sections were incubated at 4 °C with monoclonal antibody against alpha smooth muscle actin, α-SMA (Abcam, Cambridge, UK) overnight at an optimal dilution of 1:100. After washing 3-times with PBS, the sections were stained with 3,3N-Diaminobenzidine Tertrahydrochloride Peroxidase Substrate. Sections were imaged using a light microscope at 400 × magnification.

### Statistical analysis

All statistical analyses were performed with the use of *R* software (version 4.3.2), and GraphPad Prism 8. The Wilcoxon test was used to determine whether there were differences between the two groups; *p* < 0.05 was considered statistically significant.

## Results

### Identification of renal fibrosis related DEGs

**Fig. 1:**
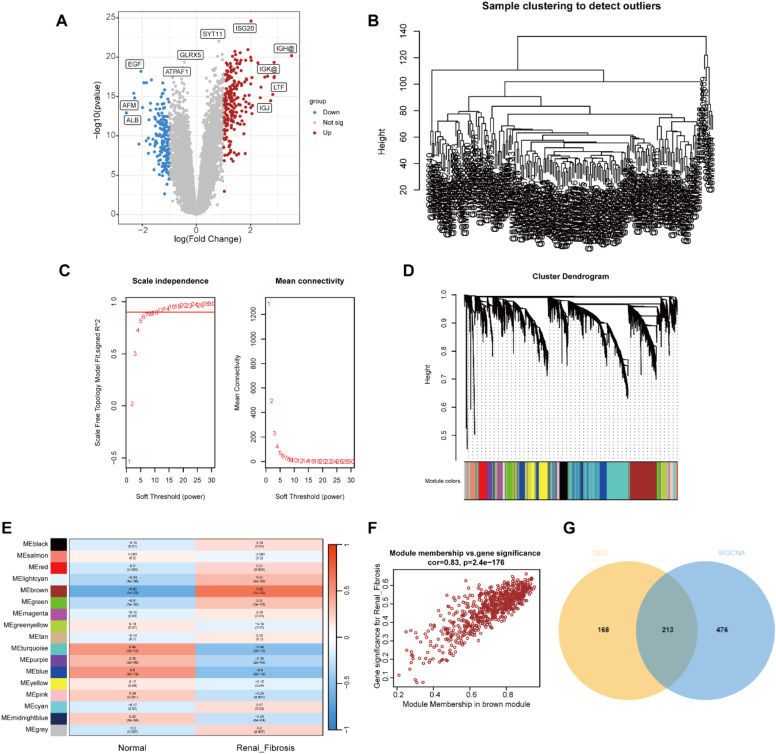
Identification of renal fibrosis related DEGs in GSE76882. (A) Volcano plot of DEGs in GSE76882. (B) Clustering dendrogram of all samples. (C) Scale-free fit index analysis and average connectivity analysis of each soft threshold power. (D) Clustering dendrogram of genes, various colors represent different modules. (E) Heatmap of the association between modules and different cluster. (F) Correlation chart between MM and GS of the clustered genes in the brown module. (G) Venn diagram showing the numbers of overlapped genes between DEGs and module genes.

First, we used the R package “limma” to screen 383 DEGs between the renal fibrosis and control group in the GSE76882 dataset, of which 230 genes were up-regulated and 153 genes were down-regulated (Fig 1A).

Next, the module genes associated with renal fibrosis were screened by WGCNA. The gene expression matrix was obtained after data preprocessing, and the abnormal samples and abnormal genes were further filtered to draw the sample clustering tree (Fig. 1B). The soft threshold was set to 12 (*R*^2^ > 0.9) to construct a scale-free network (Fig. 1C). A hierarchical clustering tree was generated using β = 12, while the clustering height of module genes was set to 0.25, and 15 gene modules were obtained for the next step of analysis (Fig. 1D). The brown module was highly positively correlated with renal fibrosis (*r* = 0.83; *p* < 0.01; Fig. 1E). In the Brown module, MM and GS were highly correlated, and 689 genes from this module were collected to be defined as module genes related to renal fibrosis (Fig. 1F). Intersecting the DEGs with the module genes yielded 213 differentially expressed renal fibrosis-related genes (Fig. 1 G).

**Fig. 2:**
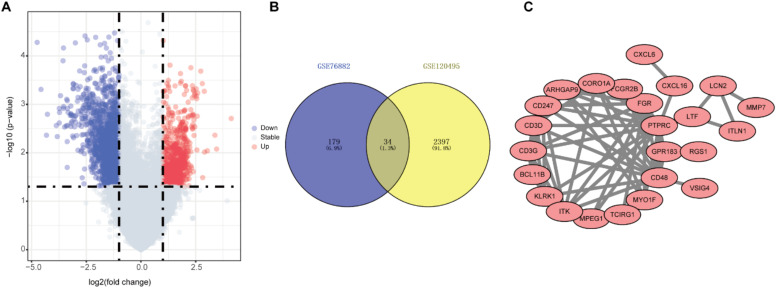
Identification of renal fibrosis related DEGs in GSE120495. (A) Volcano plot of DEGs in GSE120495. (B) Venn diagram showing the numbers of overlapped genes between GSE76882 and GSE120495. (C) Protein-protein interaction network of overlapped genes were analyzed using Cytoscape software.

Finally, in order to further eliminate the possible differences between different datasets, 2431 DEGs were screened from the GSE120495 dataset and intersected with the above results to obtain 34 genes for subsequent analysis (Fig 2A‒B).

### Functional enrichment analysis

**Fig. 3:**
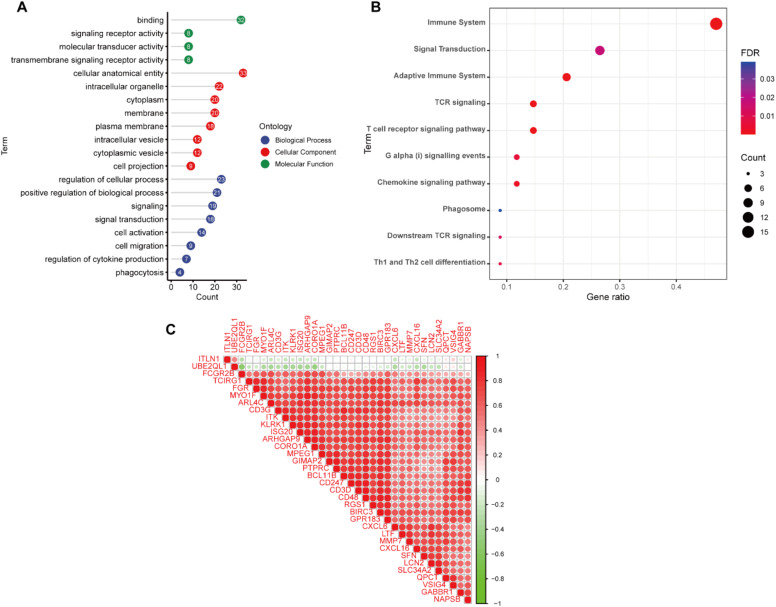
Functional enrichment analysis of renal fibrosis related DEGs. (A) GO functional analysis showing enrichment of renal fibrosis related DEGs. (B) KEGG pathway enrichment analysis of renal fibrosis related DEGs. (C) Co-expression analysis of renal fibrosis related DEGs.

The PPI network was constructed using the STRING database and Cytoscape software (Fig. 2C). Next, we explored the biological functions that might be enriched by these genes by GO and KEGG analyses. GO enrichment analysis showed that these genes were mainly enriched in signal transduction, regulation of cytokine production, cell migration and other signaling pathways (Fig 3A). KEGG enrichment analysis showed enrichment of a variety of immune-related signaling pathways, including T-cell receptor signaling pathway, chemokine signaling pathway, Th1 and Th2 cell differentiation (Fig. 3B). These results suggest that the occurrence of renal fibrosis is related to the immune response. In addition, correlation analysis revealed that ITLN1 and UBE2QL1 were negatively correlated with other genes, while the other genes were all strongly positively correlated with each other (Fig. 3C).

### Identification of the diagnostic markers for renal fibrosis

**Fig. 4:**
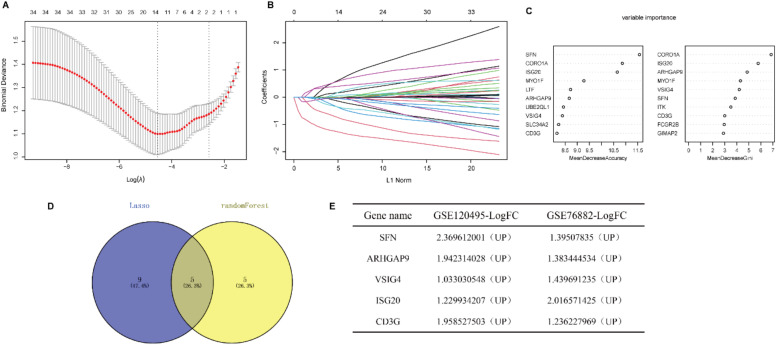
Identification of the diagnostic markers for renal fibrosis. (A) Cross-validation to select the optimal tuning parameter log (λ) in LASSO regression analysis. (B) LASSO coefficient profiles of candidate biomarkers. (C) Bubble diagram of the top 10 importance genes in the renal fibrosis model. (D) Venn diagram of intersection of genes screened by two machine learning algorithms. (E) Expression trends of overlapping genes in GSE120495 and GSE76882 datasets.

To further narrow the range of intersection genes, we used the LASSO regression algorithm to constrain the model by introducing penalty coefficients and performed 10-fold cross-validation. 14 genes were included as candidate biomarkers (Fig 4A‒B). In addition, 10 diagnostic genes were identified by the Random Forest algorithm (Fig. 4C). Five genes overlapped by the Venn diagram, and all five genes were up-regulated in renal fibrosis compared to controls (Fig. 4D‒F).

### Diagnostic power of biomarkers

**Fig. 5:**
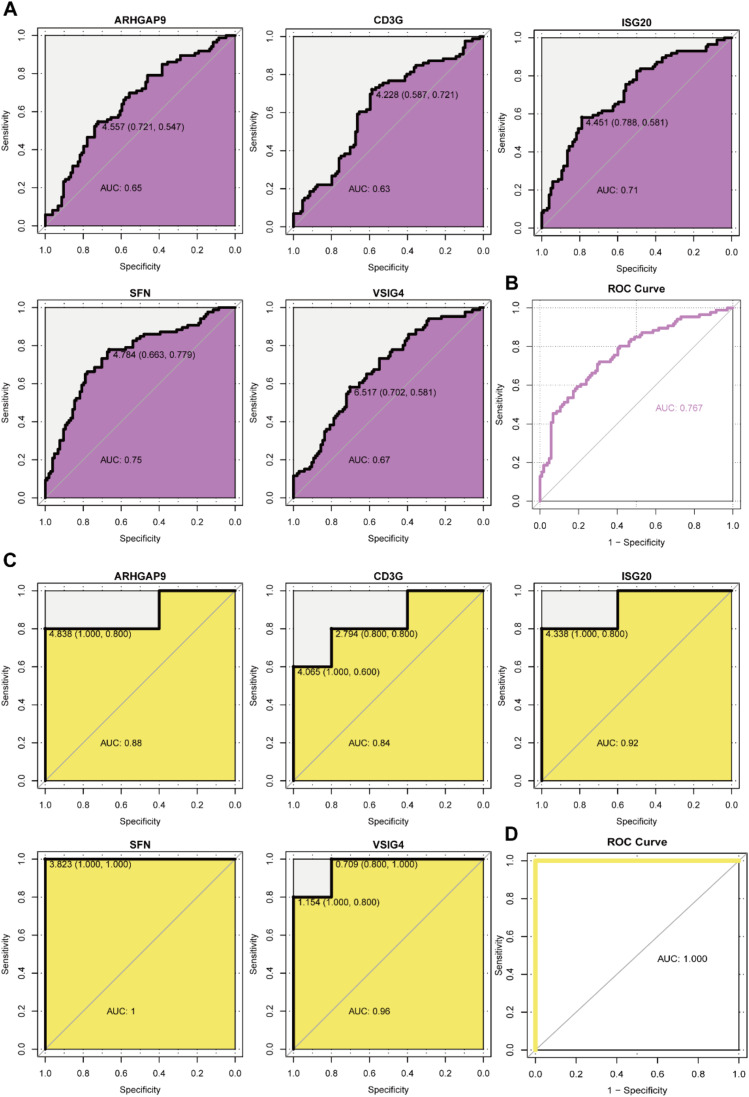
The AUCs of 5 candidate biomarkers. (A) The AUCs of 5 candidate biomarkers in the GSE76882 data. (B) The combined AUC of 5 candidate biomarkers in the GSE76882 data. (C) The AUCs of 5 candidate biomarkers in the GSE120495 data. (D) The combined AUC of 5 candidate biomarkers in the GSE120495 data.

**Fig. 6:**
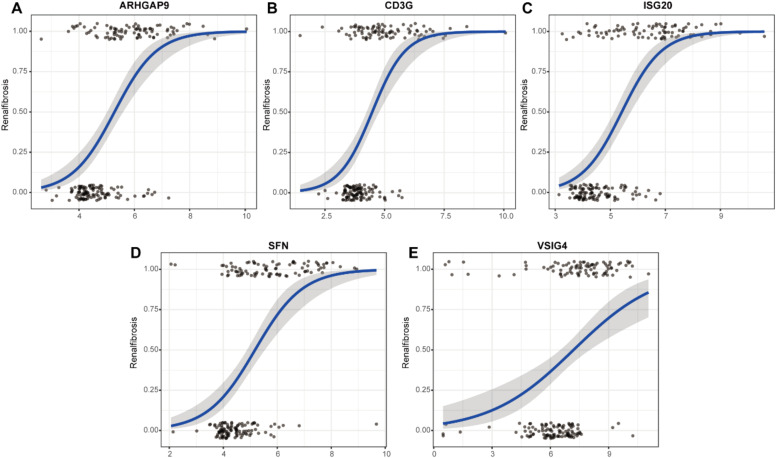
The relationship between 5 biomarkers and renal fibrosis using the method of binomial logistic regression for generalized linear models.

To evaluate the predictive role of biomarkers, in the GSE76882 dataset, the ROC curve showed that ARHGAP9, CD3G, ISG20, SFN, and VSIG4 had high specificity and sensitivity for renal fibrosis, with AUC values of 0.65, 0.63, 0.71, 0.75, and 0.67, respectively, and the combined AUC value of the five genes was 0.767 (Fig 5A‒B). In the GSE120495 dataset, the ROC curve showed that ARHGAP9, CD3G, ISG20, SFN, and VSIG4 also had high specificity and sensitivity for renal fibrosis, with AUC values of 0.88, 0.84, 0.92, 1, and 0.96, respectively, and the combined AUC value of the five genes was 1 (Fig. 5C‒D). In the GSE76882 dataset, the relationship between biomarker expression and renal fibrosis was investigated by binomial logistic regression of generalized linear models. The results of this model suggest that the relationship is monotonic. Meanwhile, the risk of renal fibrosis increases with increased biomarker expression (Fig 6).

### VSIG4 expression increased in UUO-induced renal fibrosis

**Fig. 7:**
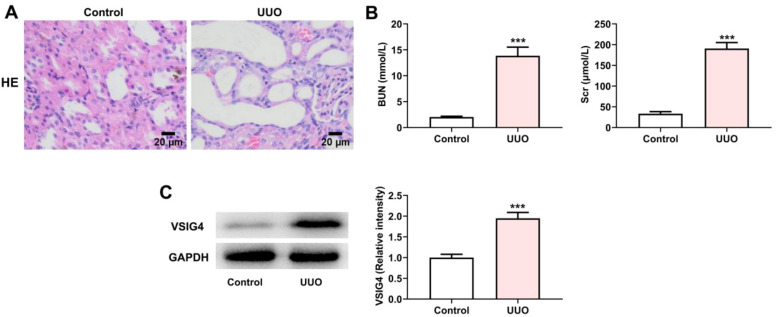
VSIG4 expression in UUO model. (A) The pathological changes of renal tissues in UUO model and control group were analyzed by H&E staining. (B) Protein levels of BUN and Scr measured by western blot assay. (C) Protein levels of VSIG4 measured by western blot assay. *** *p* < 0.001 vs. control.

To investigate the expression of VSIG4 in renal fibrosis, we first constructed a UUO mouse model as described previously. HE-staining was used to detect the pathological changes of the kidney. The results showed that the renal tubular epithelial cells in the UUO model group were detached into the lumen, the proximal renal tubular structure was absent, and the distal renal tubules were dilated (Fig 7A). The assessment of renal function in clinical practice mainly relies on the assessment of Scr and BUN levels.^15^ The levels of Scr and BUN were significantly increased in the UUO model group, indicating that UUO caused renal injury (Fig. 7B). Meanwhile, western blot results showed that VSIG4 expression was increased in UUO-induced renal fibrosis (Fig. 7C).

### Lv-shRNA-VSIG4 inhibited UUO-induced renal injury

**Fig. 8:**
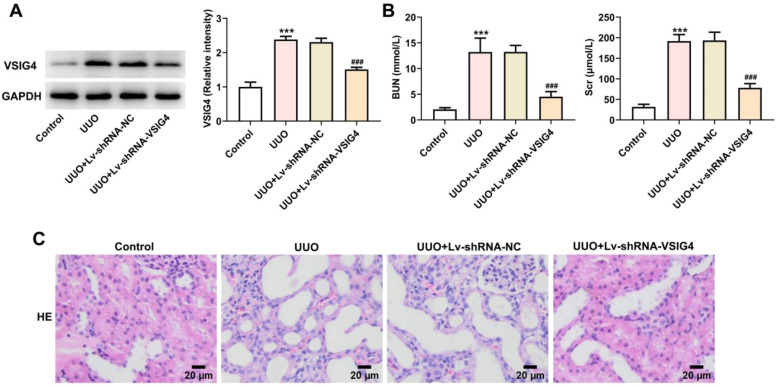
Effect of Lv-shRNA-VSIG4 on renal function injury and renal tissue pathology. (A) Protein levels of VSIG4 measured by western blot assay. (B) Protein levels of BUN and Scr measured by western blot assay. (C) The pathological changes of renal tissues were analyzed by H&E staining. *** *p* < 0.001 vs. control. ^###^*p* < 0.001 vs. UUO + Lv-shRNA-NC.

To further explore whether VSIG4 overexpression can cause renal injury and pathological changes in kidney tissue, we knocked down the expression level of VSIG4 by lentiviral particles. Western blot results showed that the expression level of VSIG4 was decreased after lentivirus interference (Fig 8A). At the same time, Scr and BUN levels were also significantly decreased (Fig. 8B). The pathological changes of the kidney were evaluated by HE-staining. Renal tissue in the UUO model showed inflammatory cell infiltration and cell degeneration, and Lv-shRNA-VSIG4 significantly inhibited renal function damage induced by UUO (Fig. 8C).

### Lv-shRNA-VSIG4 inhibited UUO-induced renal fibrosis

**Fig. 9:**
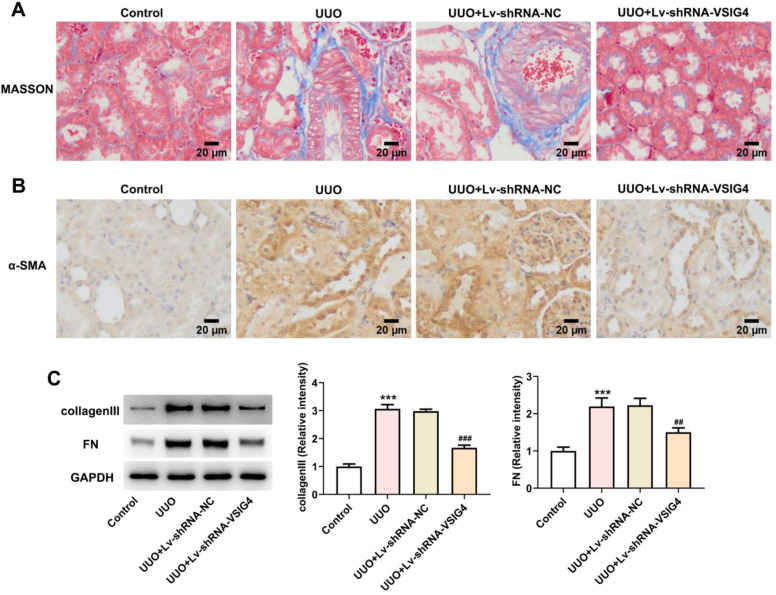
Effect of Lv-shRNA-VSIG4 on renal fibrosis levels. (A) MASSON staining of kidney tissue. (B) The expression of α-SMA in renal tissue was detected by immunohistochemistry. (C) Protein levels of collagenlll and FN measured by western blot assay. ***p < 0.001 vs. control. ^##^*p* < 0.01, ^###^*p* < 0.001 vs. UUO + Lv-shRNA-NC.

To gain insight into the potential role of VSIG4 in renal fibrosis, masson staining was used to observe the level of renal tissue fibrosis. The level of fibrosis was increased in the UUO model, showing a large number of blue-stained fibers, while renal fibrosis was significantly reduced in the Lv-shRNA-VSIG4 group (Fig 9A). α-SMA is a central feature of renal fibrosis, and its up-regulation is a typical event leading to renal fibrosis.^16^^,^^17^ The expression of α-SMA in renal tissue was detected by immunohistochemistry, which was increased in UUO induced mice, but was significantly inhibited after VSIG4 interference (Fig. 9B). In addition, the expressions of renal fibrosis-related cytokines FN and collagen III were detected by western blot. The results showed that the expression levels of FN and collagen III were increased in the UUO model group, but significantly decreased in the Lv-shRNA-VSIG4 group (Fig. 9C).

## Discussion

Although the clinical etiology of chronic kidney disease differs, each case is associated with a destructive effect of fibrosis. Renal fibrosis, the accumulation of scarring in the renal parenchyma, represents a common final pathway in almost all chronic and progressive kidney diseases.^18^ The deposition of fibrotic matrix after injury may initially contribute to the tissue repair process after mild injury, and subsequently be removed during tissue repair.^19^^,^^20^ However, during the chronic injury that occurs in CKD, fibrotic matrix deposition continues to go uncontrolled, ultimately disrupting organ architecture, reducing blood supply, and interfering with organ function.^21^ Fibrosis reduces tissue repair capacity and ultimately leads to renal failure.^22^ At present, renal biopsy is still the main clinical diagnostic method for renal fibrosis.^23^ Renal biopsy is invasive, analyzing less than 1 % of renal tissue, and there are limitations in the diagnosis of renal fibrosis.^24^ Therefore, it is very important to find a diagnostic marker that can reflect the degree of renal fibrosis.

The main characteristic of renal fibrosis is the excessive deposition and assembly of the Extracellular Matrix (ECM). Increased ECM deposition leads to changes in the chemical and mechanical environment within the tissue, which alters cellular function and exacerbates renal fibrosis. Notably, remodeling of the basement membrane and interstitial space can lead to dysfunction of the renal system.^25^ ECM proteins are assembled into a scaffold-like structure by surrounding renal cells. In turn, cells bind to this nascent ECM, leading to changes in signal transduction and cell behavior, which in turn exacerbate ECM assembly.^25^^,^^26^

In this study, we first screened DEGs between the renal fibrosis and control group, and then screened and obtained renal fibrosis-related module genes by WGCNA analysis. Combining these two results, 34 DEGs related to renal fibrosis were obtained. These DEGs were mainly enriched in immune-related signaling pathways such as cytokine regulation and the T-cell receptor signaling pathway. In particular, signal transduction promotes communication between cells and the ECM and regulates the recruitment and activation of immune cells, which is crucial for fibrosis.^27^ Cytokine regulation is involved in the regulation of inflammation and fibrosis.^28^ The T-cell receptor signaling pathway plays a pivotal role by activating T-cells that secrete profibrotic cytokines like TGF-β and IL-10, which stimulate fibroblast proliferation and ECM synthesis.^29^ The chemokine signaling pathway directs the migration of immune cells such as macrophages and T cells to sites of renal injury, promoting sustained inflammation and fibrosis.^30^^,^^31^ Furthermore, the differentiation of Th1 and Th2 cells regulates the balance between pro-inflammatory and anti-inflammatory cytokines, shaping the fibrogenic milieu.^32^ These results indicate that the occurrence of renal fibrosis is closely related to the immune response, and immune pathways not only promote inflammatory responses, but also promote ECM deposition by activating fibroblasts, thereby exacerbating the fibrotic process. These pathways collectively mediate immune cell recruitment, activation, and crosstalk with resident renal cells, facilitating fibroblast activation and excessive ECM deposition.

Then, two machine learning algorithms were used to screen potential diagnostic biomarkers for renal fibrosis. Finally, five overlapping genes were selected and defined as feature genes. Among them, VSIG4 has been widely reported as a key regulator in fibrosis across various diseases, although its specific mechanism in renal fibrosis remains unclear. Studies have shown that VSIG4 promotes the EMT of renal tubules through the downstream effect of TGF-β activation induced by high glucose,^33^ and Epstein-Barr virus promotes the EMT of renal tubular epithelial cells by inducing VSIG4.^34^ VSIG4 up-regulates the production of IL-10 and TGF-β, promoting the differentiation, migration and conversion of myocardial fibroblasts to myofibroblasts in the infarct area.^35^ Additionally, studies on pulmonary fibrosis and cardiac fibrosis suggest that VSIG4 may play a role in regulating immune cells, such as macrophages, which are critical in the fibrotic process.^35^^,^^36^ Compared with the control group, VSIG4 protein in urine and kidney tissue of UUO mice is significantly increased,^37^ but whether VSIG4 is involved in renal fibrosis caused by UUO is still unknown. Therefore, we established a mouse model of UUO to explore the involvement of VSIG4 in renal fibrosis.

V-Set and Immunoglobulin domain containing protein-4 (VSIG4) is also known as Complement Receptor of the Immunoglobulin Ig superfamily molecule (CRIg). VSIG4 inhibits the development of immune-mediated inflammatory diseases by inhibiting the activation of the complement pathway or T-cells and inducing the differentiation of regulatory T-cells to maintain immune homeostasis.^38^ VSIG4 is also overexpressed in a variety of malignancies, including non-small cell lung cancer and glioblastoma, and plays a potential tumorigenic role by regulating T-cell proliferation, migration and invasion.^39^^,^^40^ In this study, VSIG4 expression was upregulated in the UUO model group and was consistent with the expression of fibrosis markers in this model. Therefore, we speculate that VSIG4 is an important mediator in the development of renal fibrosis.

In addition to VSIG4, other classifiers such as SFN, ARHGAP9, ISG20, and CD3G were identified. These genes are involved in various cellular processes, including immune regulation, cell signaling, and apoptosis. For example, SFN is involved in regulating the cell cycle and apoptosis,^41^ while ARHGAP9 plays a role in cell migration and invasion,^42^ which are critical in fibrosis. ISG20 is involved in RNA metabolism,^43^ and CD3G is a core component of the T-cell receptor complex.^44^ These classifiers may act in concert with VSIG4, influencing immune responses and contributing to the fibrotic process in renal fibrosis.

There were some limitations to this study. We used bioinformatic analysis to screen out molecular markers in renal fibrosis. However, a prospective cohort is needed to further determine its diagnostic performance. In addition, we plan to address this in future work to better understand the role of VSIG4 in the pathogenesis of renal fibrosis.

## Conclusions

In summary, we identified five key genes (SFN, ARHGAP9, VSIG4, ISG20, CD3G) that can distinguish patients with renal fibrosis from controls, making them potential biomarkers for disease diagnosis and treatment monitoring. We have provided a deeper understanding of VSIG4’s role in the progression of renal fibrosis, particularly through its involvement in immune regulation and fibrosis-associated pathways such as EMT. Furthermore, the present study suggests that the other classifiers identified may also contribute to the fibrotic process, potentially acting in concert with VSIG4 to promote renal fibrosis. These findings provide a strong basis for further research into the role of these biomarkers in the pathogenesis of renal fibrosis and their potential utility in clinical diagnostics.

## Funding

This work was supported by the project “Hunan Clinical Medical Technology Innovation Guidance Project” (Project No. 2021SK53524).

## Authors’ contributions

Fenghua Peng: Data analysis; Writing original draft.

Xubiao Xie: Data analysis; Validation.

Longkai Peng: Methodology and software.

Chen Gao: Conceptualization; Funding acquisition; Resources, writing review and editing; Supervision.

All authors read and approved the final manuscript.

**Data availability statement**

The GEO datasets used in this study are available in the GEO database (https://www.ncbi.nlm.nih.gov/geo/) with the following data accession identifiers: GSE76882 and GSE120495.

## Ethics approval and consent to participate

The data in this study were obtained from the GEO public database; ethical approval was not required, and all methods were performed in accordance with relevant guidelines and regulations. The experimental protocol was approved by the Ethics Committee for Animal Experimentation of the ZhaoFenghua Biotech Company (Nanjing, China, nº IACUC-20,230,901–1).

## Conflicts of interest

The authors declare that they have no known competing financial interests or personal relationships that could have appeared to influence the work reported in this paper.

## Appendix Supplementary materials

Image, application 1

Image, application 2

Image, application 3
