# Supplementary material for: VSIG4 as a novel immune-related diagnostic biomarker and therapeutic target in renal fibrosis
Source: Clinics (Sao Paulo). 2025 Nov 10;80:100817. doi: 10.1016/j.clinsp.2025.100817 (PMC12648971; doi:10.1016/j.clinsp.2025.100817)
Supplement: Supplementary file 1 [file mmc1.docx]

**CLINICS-D-24-00236**

**Supplementary Material**

**Supplementary Material 1** Western blots of GAPDH, VSIG4, collagenIII, FN.

**Figure 7C** GAPDH.


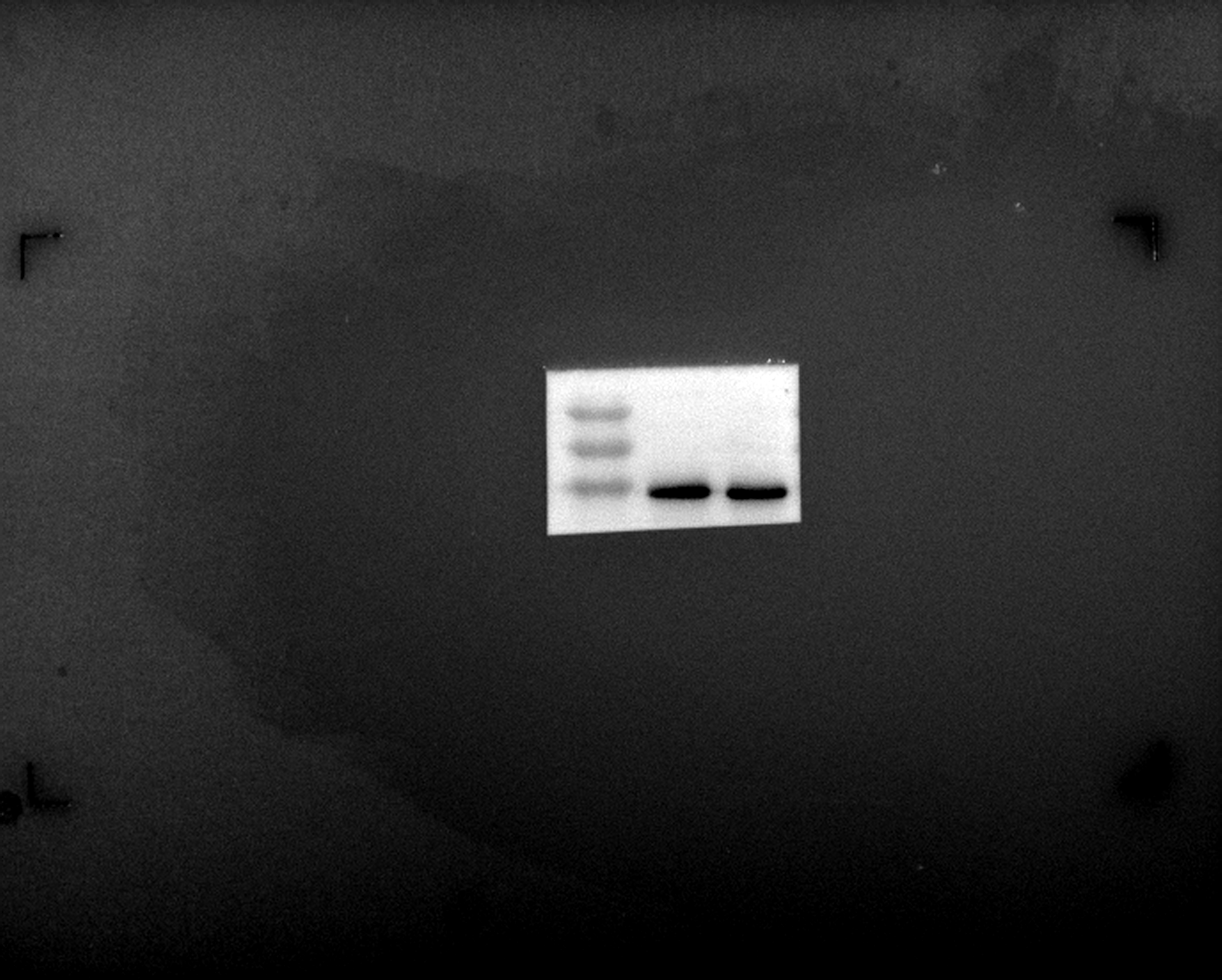


**Figure 7C** VSIG4.


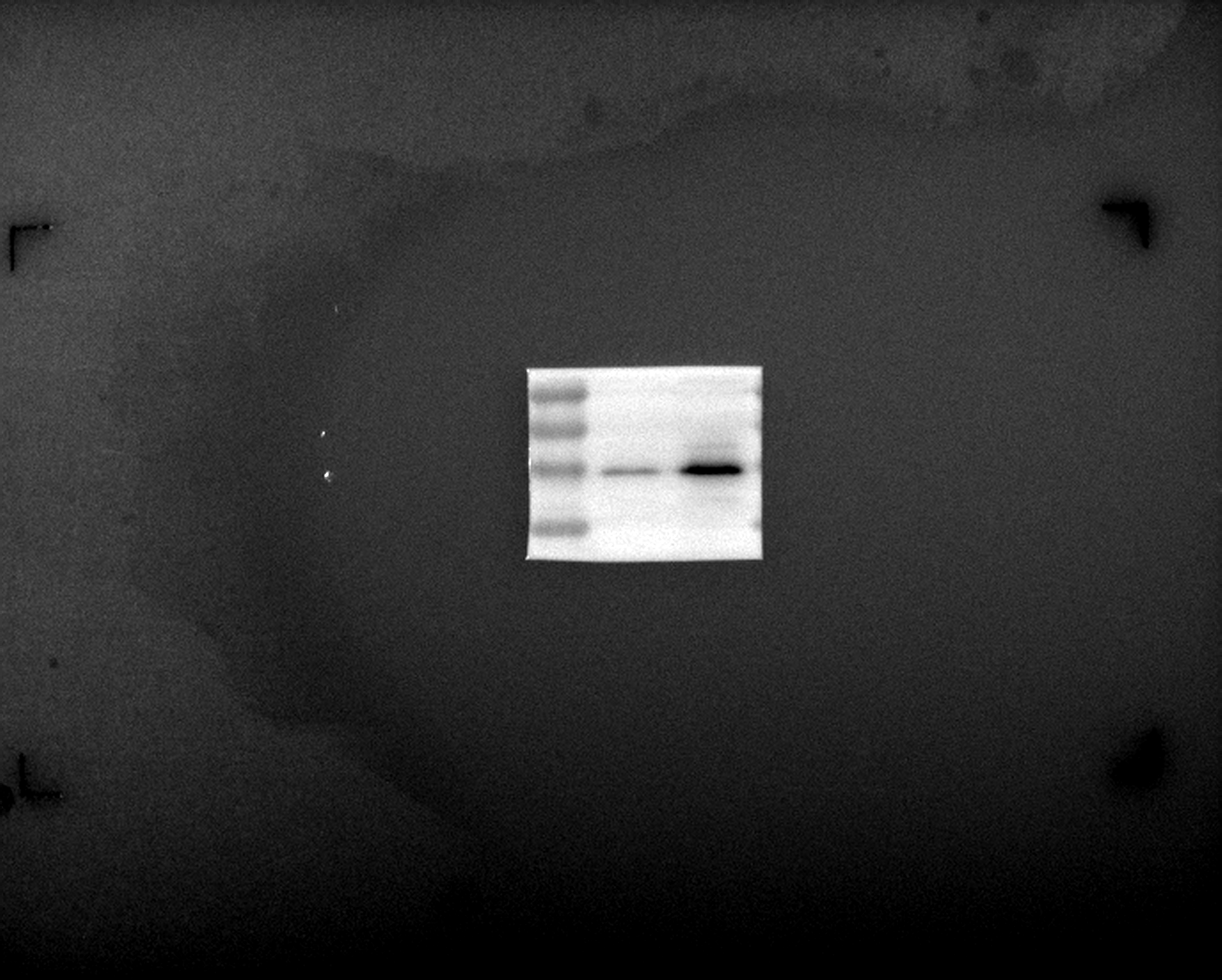


**Figure 8A** GAPDH.


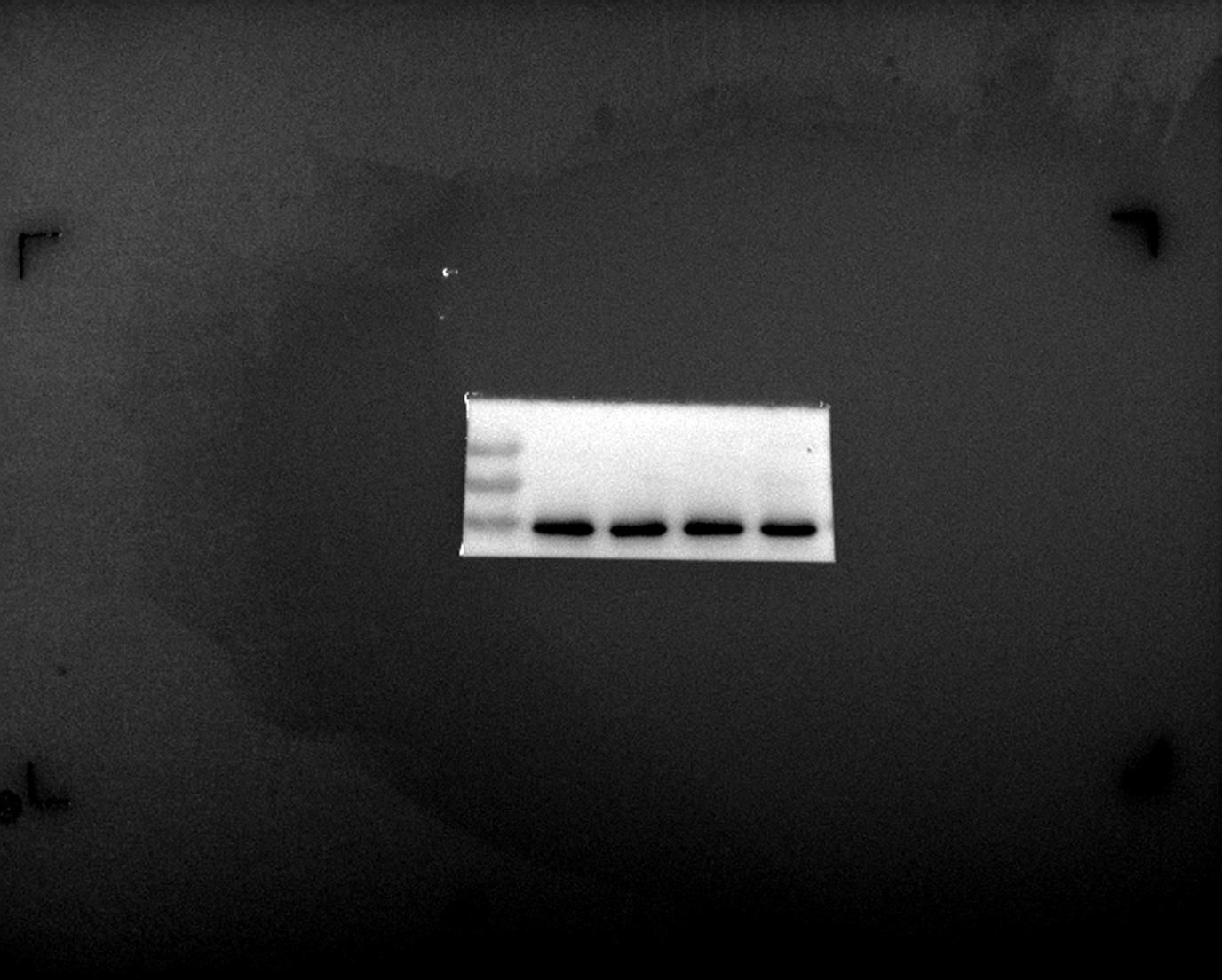


**Figure 8A** PMAIP1.


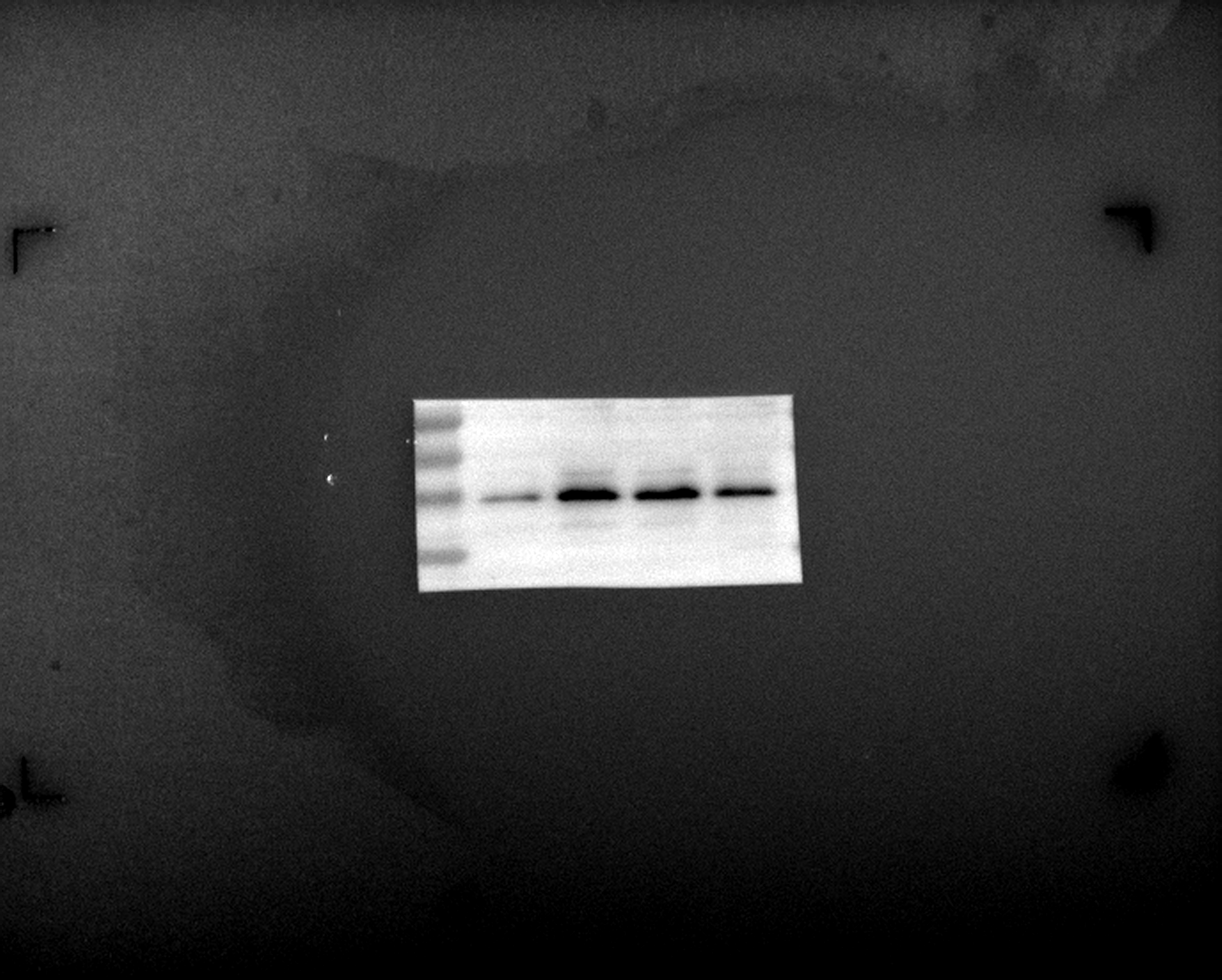


**Figure 9C** GAPDH.


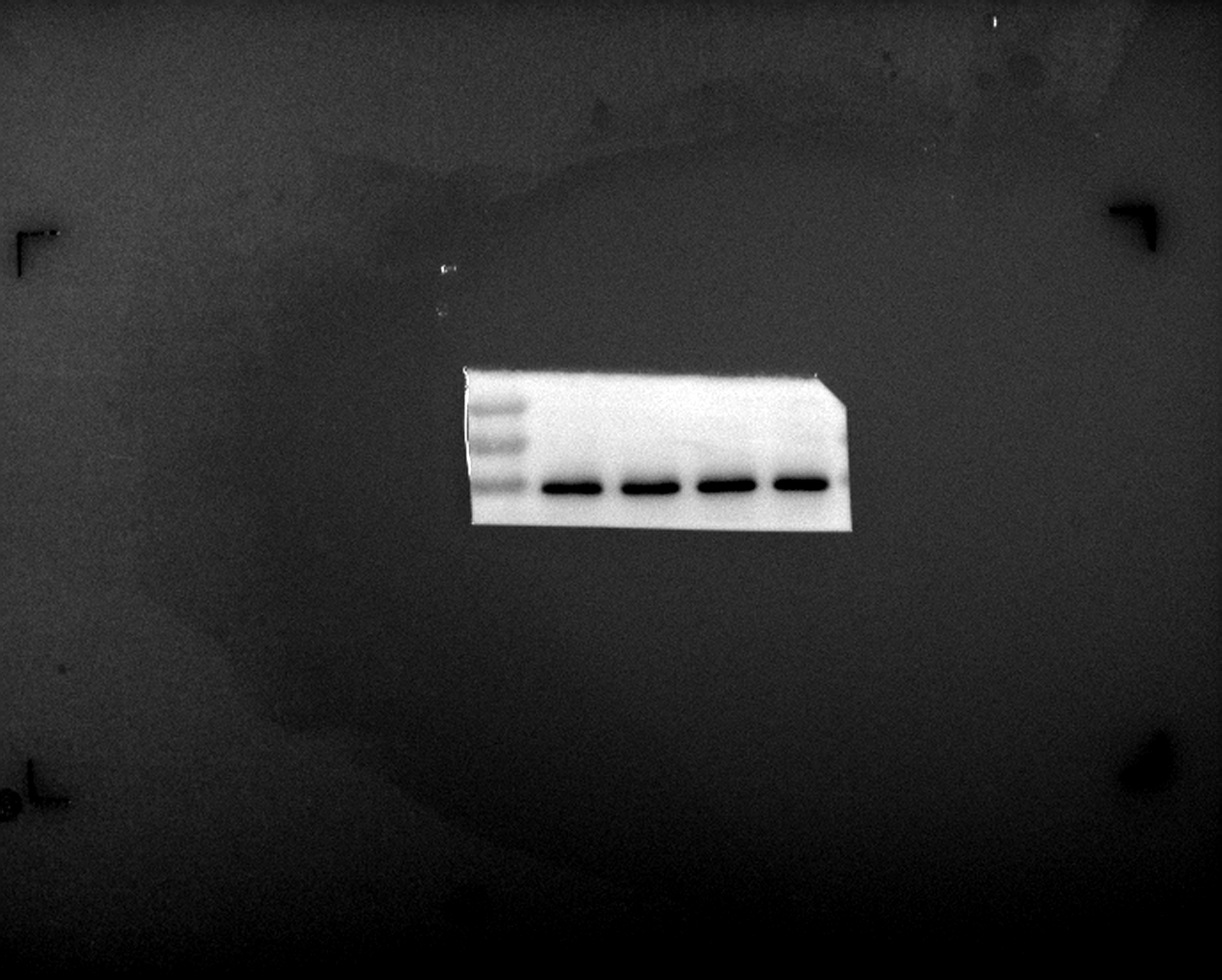


**Figure 9C** CollagenIII.


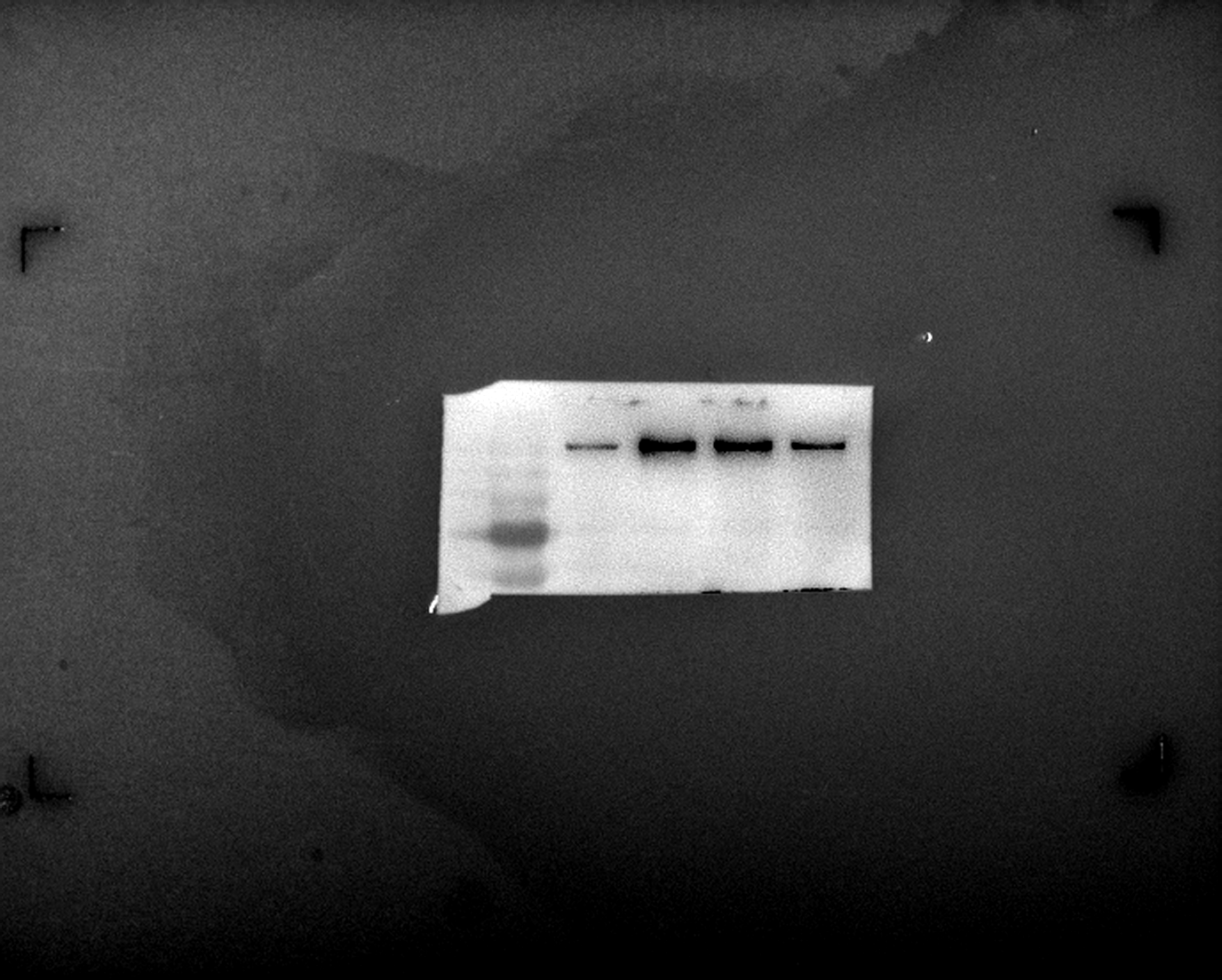


**Figure 9C** FN.


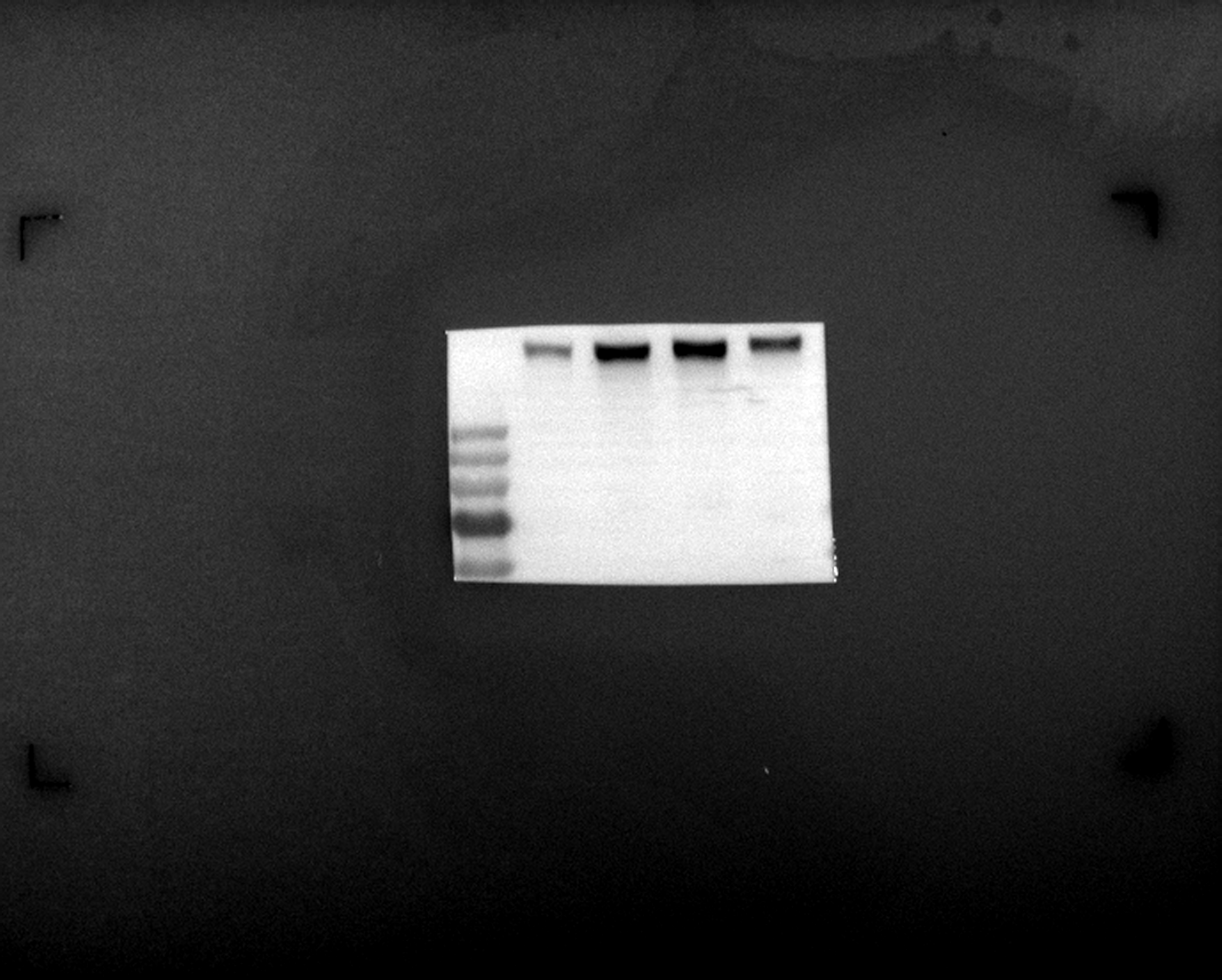


**Supplementary Table 1**

**Supplementary Table 2**
